# Ageing and remyelination failure in people with multiple sclerosis

**DOI:** 10.1093/brain/awaf373

**Published:** 2025-10-06

**Authors:** Trisha Mukherjee, Christopher E McMurran, Jonathon Holland, Cyrus Daruwalla, Gioia Riboni-Verri, J William L Brown, Robin J M Franklin, Alasdair Coles, Nick G Cunniffe

**Affiliations:** Department of Clinical Neurosciences, University of Cambridge, Cambridge CB2 0QQ, UK; Department of Clinical Neurosciences, University of Cambridge, Cambridge CB2 0QQ, UK; Department of Clinical Neurosciences, University of Cambridge, Cambridge CB2 0QQ, UK; Department of Clinical Neurosciences, University of Cambridge, Cambridge CB2 0QQ, UK; Department of Clinical Neurosciences, University of Cambridge, Cambridge CB2 0QQ, UK; Department of Clinical Neurosciences, University of Cambridge, Cambridge CB2 0QQ, UK; Altos Labs—Cambridge Institute of Science, Granta Park, Cambridge CB21 6GQ, UK; Department of Clinical Neurosciences, University of Cambridge, Cambridge CB2 0QQ, UK; Department of Clinical Neurosciences, University of Cambridge, Cambridge CB2 0QQ, UK

**Keywords:** myelin, oligodendrocyte precursor cell, magnetization transfer ratio, g-ratio, visual evoked potentials, biological ageing

## Abstract

One of the most promising strategies to delay, prevent, or reverse disability progression in multiple sclerosis is enhancing endogenous remyelination. While preclinical research has established a strong connection between ageing and remyelination failure, evidence for this same link in people with multiple sclerosis remains less secure. As clinical trials for remyelinating therapies progress, clarifying this relationship is essential. A deeper understanding could guide the selection of therapeutic candidates, refine patient selection, and optimize the timing of treatment delivery.

In this review, we describe the available evidence that has investigated the impact of age on remyelination in people with multiple sclerosis. We categorize these into pathological, imaging and clinical studies. We explore the challenges in measuring remyelination in humans and determine the implications for the connection between remyelination and age.

Current evidence suggests that there is reduced capacity for remyelination with advancing age in people with multiple sclerosis. However, these findings are at times inconsistent, and the precise contribution of ageing to remyelination failure is unclear. There does not appear to be an age cut-off beyond which remyelination is not possible, as there are pathological data supporting remyelination occurring, to some extent, across all ages. Interestingly, the impact of age may vary by lesion location. Further targeted research, specifically exploring the relationship between ageing and remyelination, is needed. With emerging evidence that ageing processes might be malleable, we conclude that targeting the biology of ageing might also be an important strategy to therapeutically enhance remyelination.

## Teaser

Mukherjee *et al.* review how ageing affects remyelination in multiple sclerosis. While most studies suggest that remyelination capacity decreases with age, findings are sometimes inconsistent, and remyelination remains possible at any age. Understanding this relationship is key to optimizing remyelination therapies.

## Introduction

Promoting endogenous remyelination decreases the vulnerability of demyelinated axons to irreversible degeneration and is therefore a leading strategy to treat disability progression in people with multiple sclerosis (MS).^1^ Remyelination is primarily achieved through a choreographed process of activation, migration, proliferation and differentiation of oligodendrocyte progenitor cells (OPCs) into newly-formed myelinating oligodendrocytes.^2^ In experimental models, each stage of this regenerative process becomes less efficient with age (reviewed by Neumann *et al*.^3^). Understanding whether ageing similarly impacts remyelination capacity in people with MS is critical for the successful development of remyelinating therapies.

**Figure 1:**
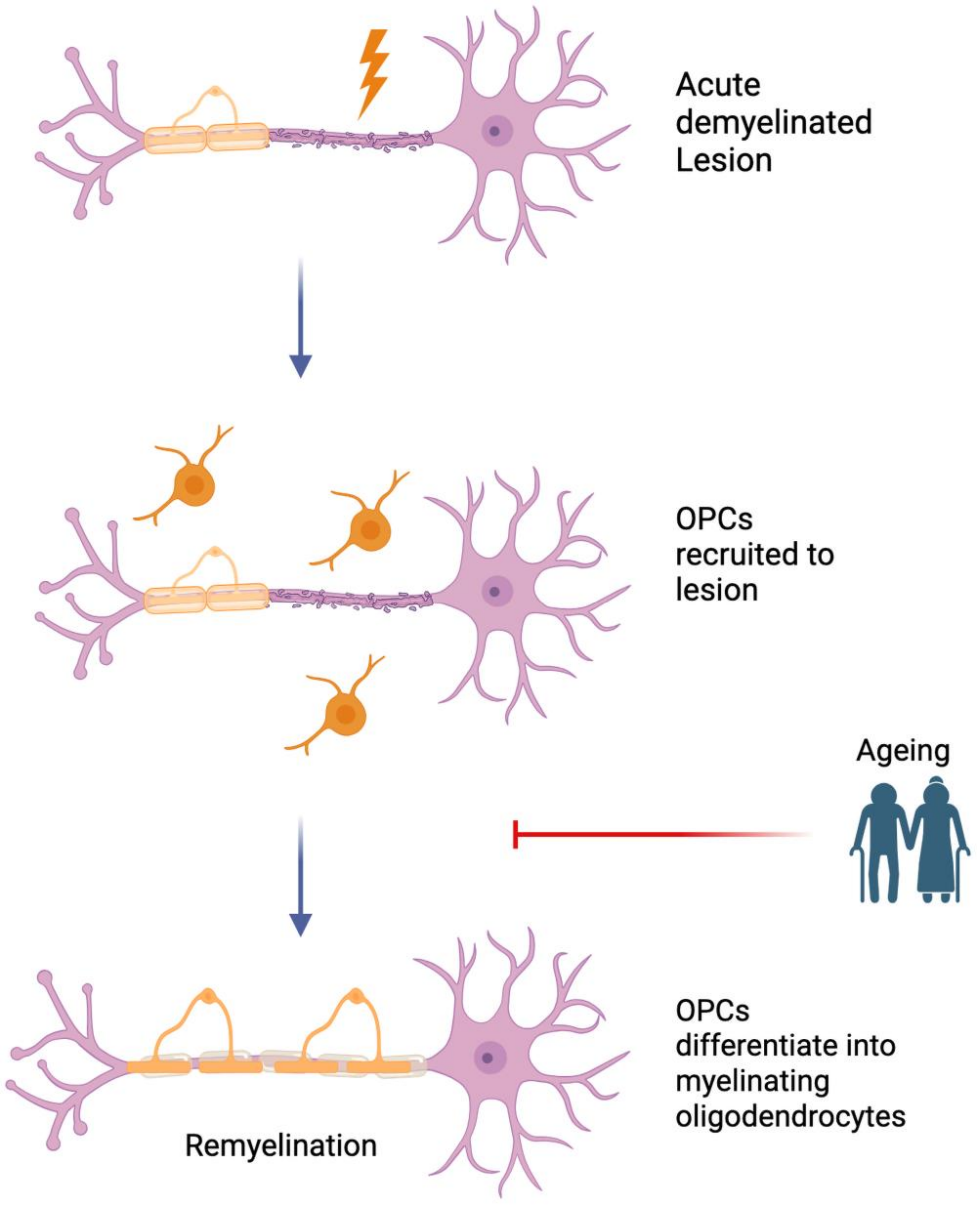
**Differentiation of OPCs into mature myelinating oligodendrocytes is the rate-limiting step for remyelination in animal models**. Created in BioRender. Karadottir, R. (2025) https://BioRender.com/usko133. OPCs = oligodendrocyte progenitor cells.

In aged rodents, differentiation of OPCs into mature myelinating oligodendrocytes has been shown to be rate-limiting for effective remyelination (Fig. 1). This was established by increasing the availability of OPCs in white matter lesions of older animals, which does not enhance remyelination.^4^ Correspondingly, OPCs from aged animals fail to differentiate even in the presence of pro-differentiation factors such as benzatropine and 9-cis-retinoic acid, which have been the basis of recent remyelination-enhancing clinical trials.^5^ Over the past two decades, studies have documented intrinsic factors within OPCs and extrinsic factors in the lesion microenvironment that are responsible for age-related deficits in remyelination.^3^ The critical role of extrinsic factors was highlighted in a heterochronic parabiosis experiment, where the remyelination capacity of older mice was rejuvenated by twinning the circulation with that of a younger mouse; exposure to the systemic environment of younger mice led to a better coordinated inflammatory response with increased OPC proliferation and differentiation in the aged mice.^6,7^ Encouragingly, the cell-intrinsic deficits in aged OPCs can be partially reversed through partial cellular reprogramming^8^ or with pharmacological treatments such as metformin.^5^ Taken together, these developments highlight potential for reversibility, raising the possibility of therapeutically enhancing remyelination throughout the lifespan.

Understanding age-dependent variation in remyelination in people with MS is more challenging. Taking a more global view of tissue regeneration, we know that across different organs in diverse mammalian species, tissue regeneration appears to universally decline with age (reviewed by Rando and Jones^9^). It is therefore to be expected that remyelination in humans would follow the same pattern. Determining the extent to which this occurs and its relevance for people with MS is crucial. It could help stratify potential remyelinating therapies to the most responsive population and optimize the design of future clinical trials for remyelinating drugs. However, there are difficulties in addressing this question, including the lack of consensus on how best to measure remyelination in humans, challenges in determining lesion age, and biases in observational studies.

To identify relevant studies for this review, we performed a systematic search of PubMed, Cochrane Library, Embase and Medline (Ovid) databases on 11 September 2024. In PubMed and Cochrane, titles and abstracts were searched using the keywords ‘age’ and ‘remyelination’. In Ovid Embase and Medline, the same keywords were searched with the option to include related terms. The search was limited to publications between 1975 and 2024. Articles were organized using the Rayyan^10^ web platform and manually screened for eligibility. Inclusion criteria were original research studies reporting remyelination data in human MS cohorts where the relationship with age had been examined. Exclusion criteria were animal studies, *in vitro* studies, non-English publications, and studies not directly addressing the research question. Additional relevant studies were identified by reference screening and expert consultation. Full search strategy and inclusion/exclusion criteria are detailed in Supplementary Table 1. For the purposes of this review, we categorize these studies into three domains: pathological, imaging and clinical.

## Pathological studies

Histological analysis of demyelinated lesions is the primary method for assessing remyelination in animal models. Similar techniques can also be applied to human MS lesions using biopsy (taken for clinical reasons) or post-mortem samples, and allow lesions to be characterized according to their inflammatory activity and degree of demyelination or remyelination.

**Figure 2:**
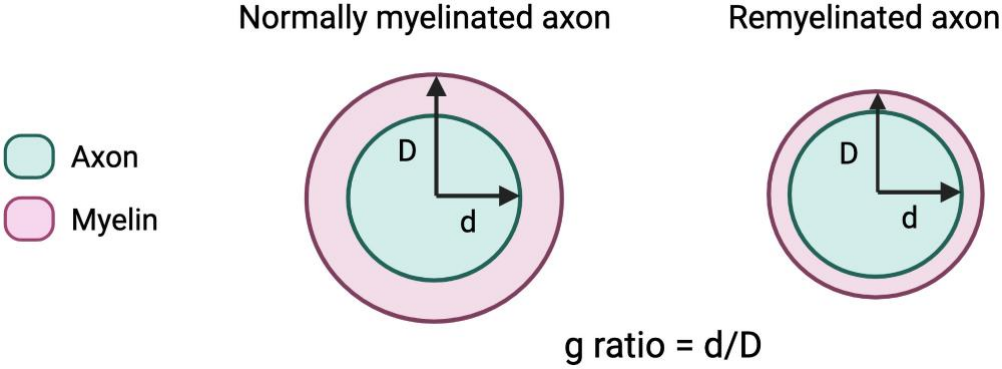
**The myelin g-ratio is higher in remyelinated axons due to the thinner myelin sheath**. Created in BioRender. Karadottir, R. (2025) https://BioRender.com/0rqq56v.

The gold standard for measuring remyelination experimentally is the visualization of a thinner myelin sheath than expected for axonal diameter, resulting in a higher g-ratio [the ratio of axonal diameter to total (axon plus myelin sheath) diameter] (Fig. 2). The increase in g-ratio following demyelination and subsequent remyelination is most evident for large diameter axons, but more difficult to appreciate for smaller axons.^2^ Changes in g-ratio can be applied to directly visualize remyelination in human MS lesions,^11^ but its utility is limited by technical factors, including the need for rapid tissue processing, and ideally *in situ* glutaraldehyde fixation with resin embedding for high magnification imaging.^12,13^ Consequently, the presence of thin myelin sheaths is typically inferred by other methods, such as by reduced staining of a myelin marker, and such areas are termed ‘shadow plaques’. Shadow plaques are generally accepted to indicate remyelination^13^; however, in some instances they may not be fully sensitive or specific to remyelinated areas.^12^

Beyond the technical pitfalls of identifying remyelination, the relationship between the age of the patient and extent of remyelination is often not the primary focus of existing pathological studies. A particular limitation is the inability to distinguish the patient’s age at the time of death (or biopsy) from their age when a specific lesion was actively undergoing demyelination or remyelination. These data are also constrained by the inability to perform longitudinal sampling, such that the appearances represent only a single time-point in the patient’s journey: typically early on in the case of biopsy, or late in the case of post-mortem. With standard pathological techniques, it is therefore not possible to make any assessment about the tempo of remyelination in any given patient. Thus, the conclusions that can be drawn from the existing pathological data regarding the relationship between age and remyelination in MS are not straightforward.

Many pathological studies examine remyelination depending on the age of the lesion, inferred by the degree of inflammation present, and these are broadly categorized into acute or chronic lesions.^14,15^ Whilst these studies may not directly assess the impact of chronological age on remyelination, they remain relevant since younger patients tend to have more acute lesions, and older patients with longer disease duration tend to have more chronic lesions.^16^ Most of the pathological studies report increased remyelination in acute versus chronic lesions. Lesions that persist and become chronic have by definition failed to remyelinate effectively^17^; it is no surprise, therefore, that chronic lesions show more limited remyelination.^18^ The observation that older patients have more chronic lesions may be in part due to remyelination failure increasing with age. However, it is also likely that these patients accumulate more lesions overall due to a typically longer disease duration. Importantly, successful remyelination requires intact axons, and therefore the extent of acute axonal loss and chronic neurodegeneration will be particularly influential to any observed remyelination.

A study looking at spinal cord lesions in patients with MS, found that chronic lesions, with fewer inflammatory cells, were associated with reduced OPC numbers.^19^ Lower OPC density was also seen in lesions from older patients, patients with longer disease duration or higher histologically-suggested lesion age.^19^ Another study, which looked exclusively at chronic lesions from patients of different ages, found that the chronic lesions from patients with longer disease duration contained fewer premyelinating oligodendrocytes.^20^ There was also a negative (but non-significant) association between the presence of premyelinating oligodendrocytes and patient age at death in this study.^20^ OPC differentiation into myelinating oligodendrocytes is a rare event in chronic MS.^21^ O4+GalC+ process-bearing oligodendrocytes are approximately 100 times less frequent than O4+GalC− OPCs in chronic MS.^21^ This reflects experimental data suggesting that remyelination fails over time in MS due to a block in OPC differentiation (Fig. 1).^4^ Independent of patient age, the extent of remyelination of chronic lesions varies by location (being more abundant in subcortical compared to periventricular areas), suggesting that local factors also influence the extent of remyelination in chronic MS lesions.^18,22^

Several studies have shown that remyelination is most extensive in acute MS lesions,^18,23,24^ and this can occur regardless of patient age or disease duration.^18^ In a post-mortem study of two people with MS in their fifties (with disease duration of over 20 years), there was a positive correlation between macrophages or microglia in the lesion border and completeness of remyelination, implying that remyelination is more effective in more inflammatory lesions, referred to as ‘early active lesions’ in this study.^25^ This is consistent with experimental data, which shows that macrophages and microglia play a key supportive role in remyelination.^26^

More recent studies have sought to provide a dynamic picture of remyelination by using biomarkers for ‘active’ remyelination, aiming to get some sense of tempo, which is impossible with traditional pathological approaches. These approaches include using markers exclusively expressed in newly formed oligodendrocytes,^27^ and using oligodendrocyte morphology,^28^ or radiocarbon dating,^29^ to indicate the age of new oligodendrocytes.

BCAS1 is a cytoplasmic protein associated with newly differentiated remyelinating oligodendrocytes. In mouse models, BCAS1+ cell density peaks during remyelination.^27^ In MS lesions, BCAS1+ cells are most abundant within actively remyelinating areas.^27^ Although BCAS1+ oligodendrocytes peak in number in the first year of postnatal white matter development, these cells are also present in cortical grey matter of older patients and in MS lesions from patients in their seventies.^27^ These cells were not restricted to lesions with high inflammatory activity and were also seen in inactive lesions, suggesting that some degree of active remyelination is possible in chronic lesions and in older patients.

Another study assessed oligodendrocyte morphology to indicate recent remyelination in a post-mortem study of 22 people with MS.^28^ Extensive remyelination of cortical lesions was seen across all ages (27–77 years), with oligodendrocyte morphology indicating that the remyelination was active across the age range. It is likely that different areas of the brain have varying remyelination capacities, and that some areas of the brain might retain the ability to remyelinate even at advanced ages, particularly cortical grey matter regions.^27,28^ Recent work has compared lesion biopsies from people with ‘normal’-onset MS (mean age at biopsy 29) to late-onset MS (mean age 69); this found that older patients have fewer ramified oligodendrocytes, suggesting a lower level of active remyelination.^30^ Similarly, BCAS1+ newly generated oligodendrocytes were more abundant in lesions from the younger patients.

Radiocarbon dating of oligodendrocytes has shown that generation of new oligodendrocytes is higher in people with more aggressive MS, who tend to be younger, have shorter disease duration, and also have more acute versus chronic lesions.^29^ This same study also highlighted a second mode of remyelination in MS lesions; from surviving mature oligodendrocytes rather than OPC differentiation.^29^ The relative susceptibility of this parallel mechanism to patient age will require further study, given it is influenced by distinct remyelination brakes.^31^

A study analysing post-mortem forebrain lesions from 51 people with MS (across mixed clinical phenotypes aged 20–75 years) found that older individuals had a higher proportion of shadow plaques than younger patients.^22^ However, interpretation of this finding is not straightforward, given the difficulties in judging the age of individual shadow plaques, as well as confounding associations between disease course and age of death. Since the time at which the lesions remyelinated is unknown, older patients, with likely longer disease duration, will have presumably accumulated more evidence of past remyelination over time than younger patients. Dying at an older age implies a milder disease course, which may have affected the extent of remyelination seen. Areas which have failed to remyelinate may shrink in older patients, increasing the relative proportion of remyelinated lesions.^22^ It has also been argued that shadow plaques might represent areas of ‘incomplete’ remyelination (with fully remyelinated lesions indistinguishable from normal tissue).^32^ Finally, shadow plaques may not always represent remyelination and can occur in the setting of axonal loss with subsequent astrocytosis.^12^

Another post-mortem study showed increased remyelination of lesions in patients with primary progressive MS (PPMS) compared with secondary progressive MS (SPMS), even though the patients with PPMS had an older median ages of onset (43–45 years) compared to those with SPMS (25–30 years), as well as older median ages of death (51–58 years and 48–52 years, respectively).^33^ From this study, disease course and inflammation appear to be important (rather than age alone) in influencing an individual’s remyelination capacity.

Finally, chronic inflammation may influence biological ageing in MS.^34^ Whereas chronological age is fixed, aspects of biological ageing are reversible, offering a pathway to restore remyelination capacity in preclinical models.^5,7^ There are an increased number of SOX2+ progenitor cells expressing p16^Ink4a^—a marker of cellular senescence—in white matter lesions of progressive MS patients compared to age-matched controls.^35^ This senescence phenotype in SOX2+ progenitor cells appears to suppress the maturation and thus myelinating capacity of OPCs *in vitro*.^35^ This effect was reversible by treatment with the mTOR inhibitor rapamycin to reduce cellular senescence, emphasizing that biological age, in addition to chronological age, may play an important role in the remyelination capacity of people with MS.

The key pathological studies to date are summarized in Supplementary Table 2. Although pathological studies provide a relatively specific and high-resolution assessment of remyelination, the invasive nature of obtaining CNS tissue, and the timely processing of this, are limiting factors, particularly for longitudinal analyses and clinical trials. The use of biopsy or post-mortem tissue may also introduce a selection bias. Those patients with MS requiring biopsy often have unusual clinical features or more severe disease, while those who die at younger ages often have more aggressive disease or significant comorbidities. Older post-mortem donors are likely to have had a more quiescent disease course, having not had early access to disease modifying therapies, which only became available in mid-1990s.

In summary, existing pathological data do not conclusively delineate the relationship between age and remyelination in people with MS. However, multiple studies show greater remyelination occurs in acute lesions which are more commonly present in younger individuals. There are data showing that remyelination occurs to some degree in older people with MS, and this may show regional variability, occurring more frequently in the cortex. Our interpretation of the pathological data is that there is significant complexity and variation in remyelination failure, which may be more dependent on age-related mechanisms in certain individuals and brain regions.

## Imaging studies

### MRI metrics of remyelination

MRI enables *in vivo* longitudinal characterization of multiple disease processes but with limited specificity. Conventional MRI sequences used to diagnose MS and monitor immunomodulatory therapy include T2-weighted, fluid-attenuated inversion recovery (FLAIR) and T1-weighted sequences (including post-contrast T1). However, the myelin specificity of these sequences is low, so they cannot reliably distinguish demyelinated from partially remyelinated lesions. For example, lesions on T2-weighted scans may exhibit different degrees of inflammation, axonal loss, demyelination and remyelination.^36,37^ A subset of T2-visible lesions are also seen on T1-weighted scans. These ‘black holes’ are more destructive, representing greater myelin but also axonal loss.^38,39^

Several advanced MRI sequences are being used as remyelination end points in clinical trials, such as myelin water fraction (MWF), magnetization transfer (MT) imaging and diffusion tensor imaging (DTI). While several myelin-sensitive MRI sequences have been validated histologically, it remains uncertain which is optimal for use in clinical studies.^40,41^

MWF is derived from multi-component T2 relaxometry and quantifies the proportion of signal attributable to water trapped between the lipid bilayers of myelin (‘myelin water’) relative to the total water signal.^42,43^ Histological validation in both human tissue and animal models has demonstrated that MWF corresponds closely to myelin content.^44,45^

MT imaging captures signal from protons bound to macromolecules (such as in myelin and cell membranes),^46^ and is usually expressed as a ratio (MTR). MTR correlates with myelin intensity in animal models^47-50^ and post-mortem MS tissue.^36,51,52^ However, MTR is not specific for myelin, also correlating with axonal count, inflammation and glial density.^51^ MTR is the most widely used imaging outcome in remyelination trials.^1^

DTI assesses the movement of water molecules in tissues, providing information about tissue microstructure.^53^ Fractional anisotropy is a measure of the directionality of water movement, i.e. whether it occurs evenly in all directions (low anisotropy) or mostly in one direction (high anisotropy), and is usually high in white matter and lower in grey matter.^54^ Combined MRI and histopathology studies have shown that both of these measures are affected by myelin content and, to a lesser degree, axonal count in post-mortem white matter brain tissue.^55^ One of the restrictions of DTI is that it cannot correctly represent fibres travelling in multiple directions in a region such as ‘crossing fibres’ in the corpus callosum.^56^ Fixel-based analysis (FBA) overcomes this by capturing individual fibre populations within a voxel, known as a fixel, to provide fibre-specific metrics.^56^ Neurite orientation dispersion and density imaging (NODDI) is another common method to model diffusion imaging, which allows mircrostructural assessment of neurites and thus may be helpful in measuring remyelination.^57^ To the best of our knowledge, neither FBA nor NODDI have yet been used in the context of directly measuring remyelination in people with MS.

MRI-derived aggregate g-ratio estimates are a promising non-invasive tool which may allow us to measure *in vivo* correlates of the histological gold-standard g-ratio longitudinally.^58,59^ The MRI-derived aggregate g-ratio is calculated at the voxel-level assuming a constant g-ratio within a voxel, and does not represent an individual fibre, unlike the pathologically derived g-ratio.^60^ Studies of its use in the context of remyelination trials would be beneficial.

Remyelination in imaging studies is generally inferred by dynamic changes in myelin content over serial scans. In order to put such dynamic changes into context, the changes of myelination patterns of the brain with age in health must first be appreciated. MWF has been used to demonstrate that myelin increases during the third decade of life, reaches a maximum plateau in the fifth decade and then decreases thereafter, with a quadratic ‘inverted U shape’ relationship between age and myelin water fraction.^42,61^ In specific anatomical areas, ageing positively correlates with myelin water fraction, including in the corpus callosum, splenium, internal capsule, external capsules and fornix.^42,62^ However, these findings have been inconsistent between studies, with another study using myelin water fraction showing a negative correlation between age and some of these brain regions, including the splenium and genu of the corpus callosum.^63^

### Remyelination imaging in paediatric multiple sclerosis

In a cohort study of 19 adolescent patients with relapsing-remitting MS (RRMS), MTR was used to assess lesion recovery following a clinical relapse.^64^ The study found that MTR recovery in acute MS lesions decreases with age during adolescence. There was a significant reduction in MTR recovery between the ages of 16 and 20 years, with older patients approaching typical recovery levels for adult-onset MS. This could be due to an overlap with the tail end of developmental myelination in adolescents, or other developmental factors, which might contribute to enhanced remyelination following an acute demyelinating event.^64^ Another paediatric imaging study using standard MRI T2 sequences showed that children have a lower proportion of supratentorial lesions than adults.^65^ These supratentorial regions would still be undergoing active primary brain myelination during adolescence, in contrast to the brainstem and cerebellum. This may suggest that areas with more mature myelin are disproportionately targeted in MS, or that remyelination capacity is improved in brain regions where primary myelination is still occurring, or both.

Quantitative susceptibility mapping (QSM), derived from T2*-images, quantifies magnetic susceptibility in biological tissues, thereby correlating with myelin and iron content.^66^ In adults, QSM iso-/hypo-intense lesions corresponded to remyelinated white matter lesions, when validated histologically using Luxol fast blue staining and immunohistochemistry against myelin basic protein.^66^ Unlike adults, all paediatric patients exhibit these iso-/hypointense remyelinated white matter lesions.^66,67^ The proportion of iso-/hypointense remyelinated lesions is higher in children (68%) compared with that reported in adults (3%–32%).^66,67^ This suggests high capacity for remyelination in paediatric MS, although the studies did not directly compare children with adults and did not see any change in the state of the lesion over time, thus not capturing any dynamic changes in myelination status.

### Remyelination imaging in adults with multiple sclerosis

The first MWF correlate of therapy-induced remyelination was seen in a *post hoc* analysis of the ReBUILD clinical trial (NCT02040298) MRI data. Patients treated with clemastine had increased MWF values in the normal appearing white matter of the corpus collosum, although it was not specified whether age had any impact on this effect.^68^

MT imaging has also been used to evaluate age-related response to remyelination-promoting drugs. The CCMR-one placebo-controlled phase 2 trial of bexarotene (ISRCTN14265371) showed statistically significant treatment effects on the MTR signal in cortical grey matter, deep grey matter and brainstem lesions, when lesions were subdivided by these locations.^69^ This treatment effect was lowest near brain surfaces, increasing with distance from the CSF.^70^ A further *post hoc* analysis of this trial found that the MTR signal in deep grey matter lesions improved only up to the age of 43 years.^71^ However, cortical grey matter lesions did not show this trend, which aligns with the pathological literature indicating that cortical grey matter lesions remyelinate well into later life.^27^

A study investigating cortical demyelination and endogenous remyelination using MTR over 5 years in 140 patients with MS (mean age of 38.2, standard deviation 12.2 years) found that cortical remyelination was extensive in half the cohort but that this occurred independently of age, disease duration or clinical phenotype.^72^ This study raises the question of whether people may inherently be ‘good or bad remyelinators’ based on variables not limited to age and further supports pathological data that indicate that remyelination in the cortex specifically may occur at all ages.^27^

High resolution imaging with 7 T MRI has shown that patients with persistent-rim lesions which failed to remyelinate were older than those with transient-rim lesions (mean ages 44.3 and 34.7 years, respectively; total 17 patients).^73^ Another study using 7 T MRI in post-mortem tissue (four MS patients) and *in vivo* (25 MS patients) found that older age ‘at time of lesion formation’ was associated with reduced remyelination.^74^ This study distinguished between remyelinated, demyelinated and mixed demyelinated/remyelinated lesions, which correlated with histopathologically assigned lesion status.

Chi-separation, another post-processing model derived from T2*-images, may better distinguish myelin and iron content.^75^ This was recently used in a study of 168 MS patients, with 108 followed up over a median of 2 years.^76^ The absolute improvement in the myelin biomarker in white matter lesions was larger in patients of a younger age, and these changes also correlated with stable or improving Expanded Disability Status Scale (EDSS) scores. This was interpreted as evidence of greater remyelination in younger patients.

In a study using myelin-specific PET in 20 patients with RRMS and eight healthy controls, a non-significant trend was seen that older age is associated with lower dynamic endogenous remyelination (*P* = 0.095), but significantly greater dynamic demyelination.^77^ Although PET offers the opportunity for higher specificity for myelin imaging than standard MRI, it is more challenging to implement in research settings due to the inherent radiation exposure with PET-CT and the ongoing technical and logistical challenges in optimizing PET-MRI imaging.^78,79^

### Limitations of imaging studies of remyelination

There are significant methodological differences between these studies, particularly in the sensitivities of the different methods to detect demyelination and remyelination, as well as in the cohorts of patients assessed. In longitudinal studies, it is also possible that changes in myelin metrics may be influenced by confounding factors such as inflammation, axon density and iron content^80,81^ rather than true changes in myelin status. There remain limitations and lack of consensus in measuring remyelination using existing technology.

Overall, imaging studies have underscored a decline in remyelination with age but have also highlighted heterogeneity in this process. Certain brain regions, such as the cortex, show capacity for remyelination up to advanced ages, which reflects findings from pathological data.

## Clinical studies

### Observational studies of clinical outcomes

Remyelination failure is thought to contribute to neurodegeneration and disability progression in MS.^82-84^ Clinical studies of disability outcomes, whilst not specific for remyelination, may still be informative. The challenge lies in the fact that disability is the result of multiple, often linked, pathological and compensatory processes occurring simultaneously. Without associated imaging or pathological data, we cannot draw any clear conclusions about remyelination and age from clinical observations alone. There can also be variability in assessments of disability and functional scores by clinicians,^85^ as well as the timing of when to categorize patients as SPMS.^86^

With these caveats acknowledged, we know that age is a major determinant of disability experienced in MS. An older age at disease onset increases the probability of PPMS,^87-89^ whilst in RRMS the risk of conversion to SPMS rises with advancing age.^90,91^ Patients tend to develop progressive disease around the fifth decade of life,^92^ and this age-associated accumulation of disability is at least partly uncoupled from whether people initially have a progressive or relapsing-remitting course.^91,93^ Although older individuals progress through disability milestones more rapidly, patients with a younger age of onset do reach the milestones at an earlier age.^93,94^ However, despite reaching disability milestones at an earlier age, a longer disease duration is required for progression to SPMS in early onset MS, compared with adult onset or late onset MS.^94^ Conversion to SPMS takes approximately 10 years longer in patients with childhood onset compared with adult onset MS.^95^ This implies that both age of onset and disease duration are important determinants of disability accumulation.

Paediatric onset MS patients are more likely to experience complete physical recovery after the first clinical event, and subsequent relapses, compared with adults, and it takes longer to reach EDSS 4 in paediatric onset MS compared with adult onset.^96,97^ However, despite EDSS outcomes following relapses being better for children than adults, children may still be left with subtle cognitive deficits and experience impairment in subsequent brain development following a demyelinating event.^98,99^ Functional recovery from MS relapses declines with advancing age.^100^

One possible explanation for these clinical observations could be an increased remyelination capacity at younger ages, perhaps related to the presence of ongoing primary myelination, that is delaying—but not preventing—progressive neurodegeneration. However, there are likely to be several other age-related factors contributing to these observations, including axonal vulnerability, neuroplasticity and co-morbid conditions that impact mobility. Thus, whilst the clinical data are potentially supportive of the hypothesis that increased age may be associated with remyelination failure, further conclusions cannot be made without combining these observations with pathological or imaging techniques specific for detecting remyelination.

### Clinical trials of remyelination

In the past decade, clinical trials have generated data on how patients of different ages respond to remyelinating drugs. Remyelination in these trials is most commonly assessed using MRI and visual evoked potentials (VEPs).^101^ Additionally, optical coherence tomography and blood-based biomarkers have been used to quantify axonal degeneration secondary to remyelination failure.

VEPs are one of the most widely used biomarkers of remyelination.^101^ Following a visual stimulus, VEPs are detected from scalp electrodes, which are averaged and amplified to form a reproducible waveform. The latency between the visual stimulus and this response is dependent on the speed of conduction of the visual pathway,^101,102^ and reductions in VEP latency have been shown to follow remyelination in experimental demyelination models closely.^102,103^

When using VEP to interpret the effect of age on remyelination, we must consider the fact that VEP latency naturally increases with normal ageing,^104-106^ particularly after age 59.^107^ Thus the threshold for what is considered to be abnormal VEP latency should be age-adjusted, otherwise patients may be inappropriately recruited to remyelination trials with perceived delayed VEP latencies who in fact have normal values for their age.

The CCMR One placebo-controlled phase 2 trial (ISRCTN 14265371) of bexarotene, a non-selective retinoid-X receptor-gamma agonist, as a remyelinating drug in MS, demonstrated statistically significant improvements in the latency of the full-field pattern-reversal visual evoked potential (FF-VEP), indicating remyelination.^69^ A *post hoc* analysis revealed that bexarotene reduced FF-VEP P100 latency the most in younger patients, and appeared to have limited efficacy in patients over the age of 42 years.^71^ No significant association was seen with disease duration. Baseline VEP amplitude tended to decline with age in this population, which may reflect reduced axonal survival with increased age. This, in turn, may impact the assessment of remyelination capacity in older individuals, as poor axonal integrity may be a confounding factor.

In this way, optical coherence tomography (OCT) has been a valuable supplement to VEP. OCT does not measure remyelination but can provide a measure of axonal integrity by assessment of the retinal nerve fibre and ganglion cell layers.^108^ This is an important consideration when selecting inclusion criteria for remyelination trials, as it allows us to ensure sufficient axons are present to feasibly remyelinate. However, no clinical trial of remyelinating drugs has significantly impacted retinal atrophy. This is likely explained by short trial durations (3–12 months) relative to the background rate of ganglion cell and retinal nerve fibre layer atrophy seen in people living with MS.^109^ It seems likely that remyelination will impact RNFL thickness (as well as MRI metrics such as brain atrophy), but it may take several years for the effects of remyelination to be clinically apparent.

Although the RENEW study (NCT01721161) of opicinumab as a remyelinating therapy in acute optic neuritis did not meet its primary end point of change in FF-VEP latency at 24 weeks, a per protocol analysis showed a statistically significant effect at 32 weeks.^110^ A further primary end point analysis of subgroups of the per protocol population showed a greater treatment effect over the age of 33, which reached statistical significance.^111^ The differences seen in the age-related responses between opicinumab and bexarotene may reflect the patients having acute optic neuritis in the opicinumab study versus chronic optic neuropathy in the bexarotene study. We know from the pathological literature that acute lesions with evidence of inflammatory activity show greater evidence of remyelination.^18,23,24^ It is plausible that in older patients, where endogenous remyelination is less efficient, remyelinating drugs are more effective in the setting of acute inflammation. Younger patients may spontaneously remyelinate better following acute optic neuritis, potentially making the effects of a remyelinating drug less evident in this population. This is supported by the finding from the RENEW study that older placebo treated patients experienced a worse recovery. The optimum time in the disease process to introduce a remyelinating therapy is therefore an important consideration, in addition to the age of the patient. The ReCOVER trial (NCT02521311), which is currently investigating the effect of clemastine fumarate on acute optic neuritis, may shed further light on this issue. However, it should be noted that, in the original positive trial of clemastine there was no significant effect of age on the treatment response.^112^

The CCMR Two trial (NCT05131828) is currently investigating the combination of metformin and clemastine as repurposed remyelinating agents for MS. Metformin has been shown to reverse age-related changes in OPCs in rodent models, thought to be due, at least partly, to its effect on the AMP-activated protein kinase pathway.^5^ Another trial is investigating the impact of metformin in children with MS (NCT04121468). These trials will provide further insights into the extent to which remyelination failure in humans is due to the age-related decline of OPCs being able to terminally differentiate into oligodendrocytes.

### Biological ageing versus chronological age

There is growing interest in distinguishing biological ageing and chronological age because, whilst chronological age cannot be changed, biological ageing may be modifiable with lifestyle or pharmacological interventions. If chronological age is a limiting factor for remyelination in people with MS, it is possible that modifying biological ageing may enhance the response to remyelinating drugs.

Chronological age refers to the number of years since an individual was born, whereas measures of biological age use biomarkers to capture age-related functional changes across different tissues and organs.^113^ These biomarkers include telomere length^114^ and DNA methylation patterns (giving rise to ‘epigenetic age’).^115^

Several studies, across both blood and glial cells, have shown that people with MS have advanced measures of DNA methylation age compared with controls.^116-118^ A recent study of epigenetic age in paediatric onset MS showed that even young patients with MS have accelerated biological ageing compared with controls.^119^ The degree of acceleration in biological ageing may affect clinical outcomes in MS: Krysko and colleagues^120^ demonstrated that patients’ telomere length could predict clinical disability in MS, even when correcting for chronological age.

Biological ageing may be an additional therapeutic target with the potential to augment the effect of remyelinating therapies. An analysis of biological ageing in the CCMR One trial cohort suggested that accelerated blood- or brain-based biological ageing might impair remyelination in response to bexarotene, although these trends were not statistically significant and varied by region.^121^ Conversely, patients had a significantly younger-appearing MRI brain age following treatment with bexarotene, which was associated with remyelination in cortical and brainstem lesions, suggesting that the process of remyelination itself may improve biomarkers of brain ageing.^121^

Future incorporation of these measures into MS research, and particularly into remyelination trials, will allow us to better appreciate the relationship between biological ageing and remyelination, and how this can then be therapeutically modulated.

## Conclusion

Although no single study provides definitive proof of remyelination failure with age in people with MS, there is broad consensus across study types that endogenous remyelination does decline with age. Children may remyelinate lesions more effectively compared with adults, but direct head-to-head studies between children and adults of different ages are needed. Increased lesion age and disease duration are important confounding factors when considering the relationship between age and remyelination, and these also appear to correlate (though variably in the case of disease duration) with reduced remyelination. Emerging evidence suggests that the effectiveness of remyelination-promoting drugs may vary with age.

Across both paediatric and adult cohort studies, there remains a vulnerability to disability accumulation, which is age-related, though also influenced by disease duration, and not entirely modifiable with current treatments.^93,94,100^ Inadequate remyelination may contribute to this process. Remyelination is likely to be shaped by many factors, including genetics, epigenetics and environmental influences, of which age is likely one contributor. Since the process of ageing itself is modulated by these same factors and can also influence epigenetic processes, the interplay between ageing and remyelination in MS is likely more complex than is currently understood. Pathological and imaging studies indicate that remyelination can be achieved across all age groups, with some cases of seemingly extensive remyelination even in those with advanced age and disease duration, particularly in the cortical grey matter.^27,28,71,72^ Observed variability may reflect regional differences in remyelination capacity, methodological differences in its measurement, or intrinsic individual variation—where some people may be inherently ‘good or bad remyelinators’.

Several unanswered questions remain. Over what timescale does remyelination decline with age, and what is the tempo of this decline? How does age interact with other influences on remyelination? What are the implications of age-related remyelination failure for patients’ symptoms and disability? Targeted studies to assess the impact of chronological and biological ageing on remyelination—whilst specifically addressing the confounding factors of lesion age and disease duration—are needed. Careful distinction is required between the current age of the patient, age at disease onset, and age at lesion formation. With advances in myelin-specific imaging modalities, alongside widely used markers of remyelination such as visual evoked potentials, these investigations are feasible. Routine incorporation of these measures, together with biomarkers of biological ageing, into MS clinical trials would be highly informative. Defining the relationship between age and remyelination will enable better selection of candidates for remyelinating therapies and optimize their timing and delivery. It will also help determine whether age-related decline in remyelination is reversible, and thus amenable to pharmacological or lifestyle interventions.

## Supplementary Material

awaf373_Supplementary_Data
