# Supplementary material for: Ageing and remyelination failure in people with multiple sclerosis
Source: Brain. 2025 Oct 6;149(1):48–58. doi: 10.1093/brain/awaf373 (PMC12782174; doi:10.1093/brain/awaf373)
Supplement: awaf373_Supplementary_Data [file awaf373_supplementary_data.pdf]

Supplementary Table 1

Search strategy and eligibility criteria

**Databases searched**	PubMed, Cochrane Library, Embase (Ovid), Medline (Ovid)
**Date of search**	11 September 2024
**Search terms**	PubMed and Cochrane: *age* AND *remyelination* (titles and abstracts). Ovid Medline and Embase: *age* AND *remyelination* with related terms*.
**Timeframe**	1975–2024
**Inclusion criteria**	Original research studies; human MS cohorts; reported data on remyelination in relation to age, full text availability.
**Exclusion criteria**	Animal studies; *in vitro* studies; non-English language publications; studies not directly assessing the research question.
**Screening process**	Articles imported into Rayyan[10] for organisation and manually screened. Additional studies identified by reference screening and expert consultation.

*Ovid related terms include synonyms, acronyms, and variants of the original term(s)

Supplementary Table 2

Summary of key pathological studies that explore a relationship between age and remyelination

Citation	Design	Relevant Findings	Interpretation
**Raine et al 1993[24]**	• Tissue from 4 patients with MS (age 18-54, disease duration 8 weeks – 13 years)	▪ Remyelination observed more frequently in acute versus chronic lesions	▪ Remyelination is more common in acute lesions
**Wolswjik 2000[21]**	• 23 post-mortem brain lesions from 15 patients (age 32-82 years) with chronic progressive MS (disease duration 8-49 years)	▪ Lesions in patients with chronic progressive MS contain few newly-differentiated oligodendrocytes<br>▪ Mature oligodendrocytes that have survived demyelination are less frequent in chronic vs acute lesions	▪ OPC differentiation is rare in chronic lesions<br>▪ Surviving oligodendrocytes are lost over time in chronic lesions
**Wolswjik 2002[19]**	• Spinal cord lesions from 16 patients (age 32-82) with longstanding MS (disease duration 8-49 years)	▪ Negative correlation between oligodendrocyte precursor cell density and patient age at death as well as disease duration	▪ OPC recruitment is reduced in chronic lesions
**Chang et al 2002[20]**	• Autopsies from 10 patients with MS (age 43-69 years, disease duration 8 months - 44 years)	▪ Patients with longer disease duration had fewer pre-myelinating oligodendrocytes, with a negative correlation found between the two<br>▪ There was no correlation between degree of pre-myelinating oligodendrocytes and age at the time of death	▪ Remyelination is less common with longer disease duration
**Patrikios et al 2006[22]**	• Autopsies from 51 patients (age 20-75) with MS – study restricted to forebrain lesions	▪ Older age at death and longer disease duration were associated with greater evidence of remyelination<br>▪ No correlation between remyelination and age of onset	▪ There is more extensive evidence of prior remyelination in the brains of those who have lived longest with MS

		▪ Remyelination occurs in all disease courses including PPMS<br>▪ Less remyelination in periventricular plaques compared with subcortical or deep white matter lesions	▪ There is considerable variability in remyelination between those who died at the same chronological age<br>▪ There is regional variability in the extent of remyelination
**Patani et al 2007[25]**	• Post mortem tissue from 2 patients with MS (both aged 51, disease duration of 21 & 22 years). Lesions from the cerebellum, brainstem and spinal cord excluded.	▪ 47% of white matter lesions showed evidence of remyelination<br>▪ Extent of remyelination was positively correlated with the presence of macrophages and microglia at the lesion borders<br>▪ No regional variability of remyelination in lesions analysed	▪ Remyelination occurs in MS even at older ages and with longer disease duration<br>▪ Remyelination occurs to a greater extent in more acute active lesions
**Kuhlmann et al 2008[23]**	• 33 biopsy and 10 autopsy samples from 43 patients with MS/CIS/SPMS (disease duration 4-34 years) and 5 controls	▪ There were significantly higher numbers of cells expressing Olig2, a marker expressed strongly in OPCs, in early MS lesions, with increased macrophage and T cell infiltration, compared with chronic MS lesions<br>▪ There were similar numbers of $Olig2^+$ OPCs in both the grey and white matter	▪ Remyelination is more likely to occur in early lesions with more inflammatory activity due to increased recruitment of OPCs
**Goldschmidt et al 2009[18]**	• Early MS lesions: 52 biopsies from 51 patients (time since symptom onset 6-93 days)<br>• Chronic MS lesions: 174 lesions from 36 autopsy cases (disease duration 3-41 years)	▪ 81% of early MS lesions vs 60% of chronic MS lesions showed signs of remyelination<br>▪ Remyelination was generally heterogeneous across lesions within the same patient<br>▪ Periventricular lesions showed less remyelination than subcortical lesions<br>▪ Cerebellar lesions were mostly completely demyelinated	▪ Remyelination is more likely to occur early in the evolution of an MS lesion<br>▪ Even within one patient, remyelination capacity varies<br>▪ There is regional variability in the extent of remyelination

		▪ There was no correlation between disease duration or age / age of death and remyelination within each of the early MS and chronic MS lesion groups	
**Bramow et al 2010**[33]	• Post mortem brain and spinal cord analysis from 51 patients with MS (ages 25-58) and 12 controls	▪ Remyelination was more extensive in patients with PPMS, despite a higher median age, compared with SPMS	▪ Disease course and degree of inflammation may be more important in determining an individual's remyelinating capacity
**Chang et al 2012**[28]	▪ Post mortem brain tissue from 22 patients (age 22 to 77 years) with MS and 6 controls	▪ Cortex showed greater remyelination than white matter<br>▪ No obvious correlation between extent of remyelination and age at death<br>▪ OPC density was not reduced in cortical lesions	▪ Cortical remyelination occurs in MS even in older people with a longer disease duration
**Fard et al 2017**[27]	▪ Post mortem brain tissue from 24 patients with MS (up to age 74) correlated to pathological data from cuprizone mouse model	▪ BCAS1+ oligodendrocytes which are expressed most densely in remyelinating mouse lesions are found in cortical grey matter and in remyelinating MS lesions up until old age<br>▪ BCAS1+ cells were found not only in lesions with inflammatory and/or demyelinating activity but also in completely inactive lesions	▪ MS lesions in patients up to the age of 74 years, and with advanced disease, showed evidence of retained remyelination capacity<br>▪ The cortical grey matter has capacity for remyelination even at older ages<br>▪ Remyelination capacity of a lesion was not completely dependent on the presence of inflammation
**Nicaise et al 2019**[35]	▪ Post mortem tissue from 7 patients with PMS (age 36-69 years; disease duration 8-40 years) and 4 age matched controls	▪ Increased markers of cellular senescence in SOX2+ progenitor cells in PMS white matter lesions compared to control white matter	▪ MS can influence biological hallmarks of ageing independent of chronological age, and this effect can be targeted pharmacologically to promote OPC differentiation
**Yeung et al 2019**[29]	▪ Radiocarbon dating of oligodendrocyte nuclei isolated from normal appearing	▪ There was no correlation between age at disease onset and the	▪ Increased remyelination may be associated with the presence of a greater

	white matter in postmortem tissue of 29 patients (age 34-81 years) with MS, and compared to previously published data on 34 controls	oligodendrocyte generation rate in MS patients<br>▪ There was a higher rate of oligodendrocyte generation in patients with more aggressive disease (who also died at a younger age)	number of acute lesions, seen in patients with more clinically aggressive disease (which tends to occur in younger patients)
**Stork et al 2025**[30]	▪ Lesion biopsies done for clinical reasons from 30 patients with 'normal'-onset (mean age at biopsy 29) MS and 31 with late-onset MS (mean age at biopsy 69)	▪ Older patients with late onset MS have fewer ramified oligodendrocytes suggesting lower level of active remyelination<br>▪ BCAS1+ newly generated oligodendrocytes in NAWM are more abundant in younger patients	▪ Active remyelination is more abundant in younger patients with earlier disease onset compared with older patients with late onset disease
